# Relationship between electronic cigarette use, dual smoking habits, and psychological distress among youth in Northern Thailand: A cross-sectional study

**DOI:** 10.18332/tid/186860

**Published:** 2024-05-17

**Authors:** Chakkraphan Phetphum, Atchara Prajongjeep, Kornkan Phuengnam

**Affiliations:** 1 Department of Community Health, Faculty of Public Health, Naresuan University, Phitsanulok, Thailand; 2 Tobacco Control Research Unit, Naresuan University, Phitsanulok, Thailand; 3 Department of Community Public Health, Sirindhorn College of Public Health Phitsanulok, Phitsanulok, Thailand; 4 Institute of Nursing, Suranaree University of Technology, Nakhon Ratchasima, Thailand

**Keywords:** e-cigarettes, smoking, mental health, depression, anxiety

## Abstract

**INTRODUCTION:**

Amidst the escalating trend of electronic cigarette (e-cigarette) use and dual smoking habits among youth, understanding their potential impact on psychological well-being is imperative. Limited research has explored this relationship, particularly among youth in settings where e-cigarettes are banned. This study investigates the relationship between current e-cigarette and traditional cigarette use patterns and the presence of depression and anxiety symptoms among youth in Thailand.

**METHODS:**

Using a cross-sectional survey encompassing 3424 individuals aged 15–24 years in Northern Thailand from December 2021 to September 2022, we accessed cigarette and e-cigarette usage over the past 30 days alongside depression and anxiety symptoms over the past two weeks. Multivariable logistic regression was employed to analyze the association of these variables, adjusting for sociodemographic factors.

**RESULTS:**

Among the surveyed youths, 10.8% reported cigarette use, 6.2% utilized e-cigarettes, and 3.3% were dual users within the past 30 days. Regarding mental health, 33.96% exhibited moderate-severe to severe depression, while 52.54% experienced anxiety at similar levels. After adjusting for covariates, both current e-cigarette users and dual users exhibited heightened depression symptoms (AOR=1.80; 95% CI: 1.30–2.51, and AOR=2.30; 95% CI: 1.49–3.55, respectively) and only e-cigarette use had increased odds of increased anxiety levels (AOR=1.70; 95% CI: 1.24–2.32) compared to non-users. Notably, current cigarette smokers demonstrated no association with depression but had decreased odds of higher levels of anxiety compared to non-users (AOR=0.73; 95% CI: 0.55–0.96).

**CONCLUSIONS:**

A substantial number of Thai youths are using e-cigarettes and experiencing psychological distress. E-cigarette use is associated with heightened levels of depression and anxiety, whereas cigarette smoking is not associated with depression but is likely to be associated with an increased reporting of anxiety. These contradictory findings highlight the need for comprehensive investigations, especially when e-cigarettes are prohibited.

## INTRODUCTION

The trend of traditional cigarette use in the young population has been steadily declining^1^, while electronic cigarettes, commonly known as e-cigarettes, have witnessed an unprecedented surge in popularity^2^. In 2019, a substantial number of youths aged 15–24 years, worldwide, were estimated to be tobacco users, totaling approximately 155 million^3^. Although specific estimates are unavailable, a 2022 systematic review of global data revealed that the pooled prevalence of current e-cigarette vaping and concurrent use of e-cigarettes and traditional cigarettes among individuals aged 9–25 years was 7.7% and 4%, respectively^4^. With the growing exposure of youth to e-cigarettes, apprehensions have surfaced regarding the health effects of these novel products on young individuals.

E-cigarettes are often promoted as a safer alternative to traditional tobacco smoking, triggering a public health debate that extends well beyond their potential for harm reduction.

Despite avoiding the harmful combustion process of traditional cigarettes, e-cigarette use still leads to the thermal breakdown of liquid bases, resulting in the formation of hazardous by-products such as tobacco-specific nitrosamines, polycyclic aromatic hydrocarbons, aldehydes, metals, and volatile organic compounds^5^. While extensive evidence has reported the physical health consequences of e-cigarettes, including impacts on respiratory, cardiovascular, gastrointestinal, neurological and immune systems^6^, their mental health implications remain relatively contentious.

There exists uncertainty in various reviews regarding the mental health implications of e-cigarette use among young individuals, particularly those based on studies conducted in high-income countries with less stringent legislative policies controlling these products. For instance, a 2022 scoping review underscores that e-cigarette users are more likely to experience depression and suicide attempts compared to non-users^7^. Furthermore, a systematic review identified increased mental health problems such as depression, anxiety, suicidality, attention deficits, hyperactivity disorder, impulsivity, and perceived stress associated with e-cigarette use among youth^8^. However, the primary evidence from these reviews is limited by imprecision.

Nicotine, a commonly found addictive substance in e-cigarettes, is acknowledged for its potential impact on the developing brains of young individuals^9^. However, this also applies to smoking, which remains prevalent. Nevertheless, the evidence linking nicotine directly to mental health issues remains inconclusive, just as the overall influence of e-cigarettes on mental disorders lacks a definitive consensus.

Thailand is among the more than 30 nations that have implemented a complete ban on the sale of e-cigarettes, when traditional cigarettes are allowed and limited to those aged ≥20 years. While the prevalence of youth e-cigarette use in Thailand has been lower than the global average, as indicated by the Global Youth Tobacco Surveys conducted from 2014 to 2019^10^, recent data show a significant increase in e-cigarette usage among young people in the country^11^. This trend mirrors patterns observed in high-income countries, such as Korea^12^ and the United Kingdom^13^, where these products are legally available. These circumstances raised public health concerns regarding the potential health risks associated with e-cigarette use among the youth population.

While numerous studies have examined the impact of e-cigarettes on mental health^7,8^, causality remains undetermined. Additionally, previous research, primarily conducted in high-income countries with more permissive regulations on these products, lacks focus on regions where e-cigarettes are prohibited. It is crucial to recognize that differing legislative environments are likely to shape diverse vaping and smoking behaviors and their associated health risks. Moreover, young people are particularly vulnerable to the effects of substance use. Consequently, this study aims to investigate the correlation between vaping and smoking behaviors and the prevalence of depression and anxiety symptoms among young individuals in Thailand.

## METHODS

### Study design and sampling

This study was a cross-sectional survey conducted among individuals aged 15–24 years in Northern Thailand. The sample size for this study was determined using the infinite population proportion formula, incorporating several parameters: proportion (P)=0.10, reflecting the rate of depression among young Thai people^14^; error=0.02 (15% of P); alpha=0.05; Z (0.98)=1.96; and cluster effect=2. Initially, a total of 3046 individuals were estimated. To account for potential non-responses and incomplete responses, 15% of this number was added. Consequently, the final sample size was determined to be 3503 individuals. The sampling process followed a stratified two-stage cluster approach to ensure the selection of representative samples from Northern Thailand. Firstly, the region was divided into three strata: upper North, central North, and lower North, using a stratified random sampling method. Secondly, two provinces were randomly selected from each stratum using a cluster random sampling method, resulting in six provinces. Thirdly, another round of cluster random sampling was employed to select one secondary school, one vocational school, and one university from each province, totaling 18 educational institutions. Lastly, once the target institutions agreed to participate in data collection, representative classes were randomly chosen from each institution using a simple random sampling method (lottery method). Lists of student names from each classroom were then compiled, and random sampling was carried out until the required number of samples was obtained.

### Measures

*Exploratory variables*

Sociodemographic characteristics were evaluated through a checklist comprising questions pertaining to sex, current education level, monthly pocket money, and latest Grade Point Average (GPA). Additionally, participants were asked to provide their ages via a short-answer question. Assessment of current substance use, including alcohol and cannabis, was conducted by querying participants about their usage of these substances within the past 30 days.

*Independent variables*

Current e-cigarette vaping and traditional cigarette smoking patterns were determined by two questions: ‘In the past 30 days, have you smoked a cigarette, even 1 or 2 puffs?’, and ‘In the past 30 days, have you smoked an electronic cigarette, even 1 or 2 puffs?’. Participants who answered ‘Yes’ to both questions were categorized as ‘current dual users’, those who responded ‘Yes’ to either question were classified as ‘current users (e-cigarette or cigarette)’, and those who answered ‘No’ to both questions were identified as ‘non-users’.

*Dependent variables*

The severity of depressive symptoms was measured using the Thai version of the Patient Health Questionnaire for Adolescents (PHQ-A) with internal consistency with an alpha coefficient of 0.92^15^. This questionnaire included a 9-item checklist that inquired about various symptoms experienced over the past two weeks, such as ‘feeling down, depressed, irritable, or hopeless’, ‘trouble falling asleep, staying asleep, or sleeping too much?’ and ‘poor appetite, weight loss, or overeating’. Participants rated their responses on a 4-point Likert scale: ‘0=Not at all’, ‘1=Several days’, ‘2=More than half the days’, and ‘3=Nearly every day’. The severity of depression symptoms was categorized as minimal (0–4), mild (5–9), moderate (10–14), moderate-severe (15–19), and severe (20–27).

Anxiety symptom levels were assessed using the Generalized Anxiety Disorder-7 (GAD-7) with internal consistency with an alpha coefficient of 0.92^16^, which had been translated into the Thai language and underwent a quality check by language experts. This scale included a 7-item checklist about the frequency with which individuals had experienced specific problems over the past two weeks, such as ‘feeling nervous, anxious, or on edge’ and ‘not being able to stop or control worrying’. Participants responded on a 4-point Likert scale: ‘0=Not at all’, ‘1=Several days’, ‘2=More than half the days’, and ‘3=Nearly every day’. Anxiety severity was categorized based on the score: 0–4: Minimal Anxiety, 5–9: Mild Anxiety, 10–14: Moderate Anxiety, and 15–21: Severe Anxiety’.

### Data collection

The study involved assembling groups of target participants in common locations and providing them with comprehensive information about the study, its objectives, its importance, and participants’ rights to participate, decline participation, or withdraw at any point without providing reasons. For students aged <20 years, informed consent from parents or guardians was obtained through the schoolteachers prior to the day of data collection. Students who had obtained parental consent and submitted a signed consent form were then invited to participate in the survey and provided with the questionnaire. Participants were given detailed instructions on completing the anonymous questionnaire and assured that their responses would be treated with confidentiality. The questionnaire took a maximum of 30 minutes to complete. Following the conclusion of all data collection processes, 3424 questionnaires were completed and returned, resulting in an exceptionally high response rate of 97.8%. The data collection period spanned from December 2021 to September 2022. The study protocol received approval from the Human Research Ethics Committee of Naresuan University (Project certification No. 56 02 03 000).

### Analysis

The data analysis was conducted using SPSS version 17.0 for Windows. Sociodemographic characteristics and the participants’ current vaping or smoking status were described in terms of frequency and percentage, categorized based on their severity levels of depression and anxiety symptoms. Scale variables were dichotomized based on the average calculated. Depression and anxiety levels were categorized using established thresholds: mild/moderate (0–9) and moderate-severe/severe (10–27) for depression^17^, and minimal/mild (0–9) and moderate/severe (10–21) for anxiety^16^. Univariate analysis was employed to assess the unadjusted relationship between current vaping/smoking status, current substance use, sociodemographic characteristics, and levels of depression and anxiety.

Multivariable logistic regression with the enter method was used to explore the associations between vaping/smoking status and levels of depression and anxiety. The analyses adjusted for various factors, including sex, current education level, monthly income, GPA, past 30-day alcohol use, and past 30-day cannabis use. Hosmer-Lemeshow statistics were used to assess the model’s fit prior to conducting the multivariate logistic regression. The analysis revealed a good model fit for the model, demonstrated by the Chi-square test results (χ^2^=11.64, p=0.168, and χ^2^=9.84, p=0.277). The significance level was two-sided with a threshold of p<0.05.

## RESULTS

### Descriptive analyses

As shown in Table 1, the study involved 3424 young individuals, 56.3% of whom were females, and the average age was 17.85 years (SD=2.11). The distribution of their current education level was relatively even, with 36.1% being secondary students, 32.8% enrolled in vocational programs, and 31.1% pursuing undergraduate degrees. Their average monthly income was 2715 THB (SD=4004), and their GPA averaged 3.23 (SD=0.53). In terms of substance use, the prevalence rates for current smokers, current e-cigarette users, and dual users were 10.8%, 6.2%, and 3.3%, respectively. During the preceding 30 days, 33.1% of the participants consumed alcohol, while 10.2% engaged in the use of cannabis. In terms of mental health, the prevalence of moderate-severe to severe depression was 33.96%, while the prevalence of anxiety at the same level was 52.54%.

**Table 1:** Participant characteristics by levels of depression and anxiety, surveyed in 2021–2022, in Northern Thailand (N=3424)

*Characteristics*	*Total n (%)*	*Level of depressive symptoms*		*Level of anxiety symptoms*	
		*Mild-moderate n (%)*	*Moderate-severe to severe n (%)*	*Minimal-mild n (%)*	*Moderate-severe n (%)*
**Sex**					
Male	1498 (43.7)	1218 (81.3)	280 (18.7)	817 (54.5)	681 (45.5)
Female	1926 (56.3)	1338 (69.5)	588 (30.5)	808 (42.0)	1118 (58.0)
**Age** (years), mean ± SD	17.85 ± 2.11	17.92 ± 2.07	17.64 ± 2.21	17.98 ± 2.05	17.73 ± 2.16
<20	2589 (75.6)	1925 (74.4)	664 (25.6)	1220 (47.1)	1369 (52.9)
≥20	835 (24.4)	631 (75.6)	204 (24.4)	405 (48.5)	430 (51.5)
**Current education level**					
Secondary student	1236 (36.1)	861 (69.7)	375 (30.3)	514 (41.6)	722 (58.4)
Vocational student	1123 (32.8)	917 (81.7)	206 (18.3)	628 (55.9)	495 (44.1)
Undergraduate student	1065 (31.1)	778 (73.1)	287 (26.9)	483 (45.4)	582 (54.6)
**Monthly income** (THB), mean ± SD	2715 ± 4004	2708 ± 4110	2733 ± 3674	2694 ± 3538	2733 ± 4382
**GPA**, mean ± SD	3.23 ± 0.531	3.23 ± 0.522	3.23 ± 0.572	3.21 ± 0.521	3.24 ± 0.539
**Past 30-day alcohol use**					
No	2292 (66.9)	1733 (75.6)	(24.4)	1093 (47.7)	1199 (52.3)
Yes	1132 (33.1)	823 (72.7)	309 (27.3)	532 (47.7)	600 (53.0)
**Past 30-day cannabis use**					
No	3075 (89.8)	2291 (74.5)	784 (25.5)	1446 (47.0)	1629 (53.0)
Yes	349 (10.2)	265 (75.9)	84 (24.1)	179 (51.3)	170 (48.7)
**Past 30-day cigarette use**					
No	2728 (79.7)	2058 (75.4)	670 (24.6)	1284 (47.1)	1444 (52.9)
Exclusive traditional	370 (10.8)	287 (77.6)	83 (22.4)	213 (57.6)	157 (42.4)
Exclusive electronic	211 (6.2)	138 (65.4)	73 (34.6)	80 (37.9)	131 (62.1)
Dual (both traditional and electronic)	115 (3.3)	73 (63.5)	42 (36.5)	48 (41.7)	67 (58.3)

GPA: grade point average. THB: 1000 Thai Baht about US$27.

Data analysis (Table 1) revealed that 36.5% of dual users, 34.6% of current e-cigarette users, 22.4% of individuals who smoked traditional cigarettes, and 24.6% of those who had not used any tobacco products in the past 30 days reported experiencing moderate-severe to severe levels of depression. In terms of anxiety symptoms, a higher proportion was observed among current e-cigarette users (62.1%) and current dual users (58.3%), as well as non-current users (52.9%), while a comparatively lower proportion was found among current cigarette smokers (42.4%).

### Univariate analyses

In the unadjusted model (Table 2), e-cigarette users (OR=1.63; 95% CI: 1.21–2.19) and dual-users (OR=1.77; 95% CI: 1.20–2.61) had a higher likelihood of experiencing increased depression symptoms when compared to non-current users. Additionally, among young individuals with heightened depression symptoms, being female (OR=1.91; 95% CI: 1.63–2.25) were identified with increased odds, while attending a vocational college (OR=0.5; 95% CI: 0.43–0.63) was associated with lower odds. Concerning anxiety, traditional cigarette smokers were less likely (OR=0.66; 95% CI: 0.53–0.82), while e-cigarette users were more likely (OR=1.46; 95% CI: 1.09–1.94) than non-current users to experience increased anxiety symptoms. Additionally, those being female (OR=1.66; 95% CI: 1.45–1.90) and younger (OR=0.94; 95% CI: 0.91–0.98) were more likely to experience increased anxiety, while those attending vocational schools (OR=0.56; 95% CI: 0.48–0.66) were less likely to report experiencing increased anxiety.

**Table 2:** Multiple logistic regression analysis: e-cigarette/cigarette use and depression/anxiety symptoms among young individuals, surveyed in 2021–2022, Northern Thailand (N=3424)

*Variables*	*Moderate-severe to severe depressive symptoms*						*Moderate-severe anxiety symptoms*					
	*OR*	*95% CI*	*p*	*AOR^a^*	*95% CI*	*p*	*OR*	*95% CI*	*p*	*AOR^b^*	*95% CI*	*p*
**Female** (Ref. Male)	1.91	1.63–2.25	<0.001*	0.55	0.45–0.67	<0.001*	1.66	1.45–1.90	<0.001*	1.46	1.23–1.73	<0.001*
**Age** (years) ≥20 (Ref. <20)	0.94	0.78–1.12	0.483	0.99	0.80–1.25	0.995	0.95	0.81–1.11	0.487	0.98	0.81–1.18	0.804
**Current education level** (Ref. Secondary students)												
Vocational students	0.52	0.43–0.63	<0.001*	0.59	0.47–0.74	0.001*	0.56	0.48–0.66	<0.001*	0.63	0.52–0.76	<0.001*
Undergraduate students	0.85	0.71–1.02	0.073	0.71	0.56–0.90	0.005*	0.86	0.73–1.01	0.069	0.80	0.64–0.98	0.032*
**Monthly income**	1.00	0.99–1.00	0.874	1.00	1.00–1.00	0.882	1.00	0.90–1.01	0.780	1.00	1.00–1.00	0.617
**GPA**	1.01	0.87–1.17	0.900	0.09	0.76–1.04	0.143	1.10	0.97–1.25	0.146	0.98	0.85–1.13	0.789
**Past 30-day alcohol use** (Ref. No)	1.16	0.99–1.37	0.066	0.82	0.68–0.99	0.036*	1.03	0.89–1.19	0.703	0.92	0.78–1.08	0.279
**Past 30-day cannabis use** (Ref. No)	1.08	0.83–1.40	0.561	1.16	0.83–1.63	0.375	1.19	0.95–1.48	0.131	0.95	0.72–1.26	0.737
**Past 30-day cigarette use** (Ref. No)												
Exclusive traditional	0.89	0.69–1.15	0.371	1.07	0.77–1.50	0.688	0.66	0.53–0.82	<0.001*	0.73	0.55–0.96	0.026*
Exclusive electronic	1.63	1.21–2.19	0.001*	1.80	1.30–2.51	<0.001*	1.46	1.09–1.94	0.011*	1.70	1.24–2.32	0.001*
Dual (both traditional and electronic)	1.77	1.20–2.61	0.004*	2.30	1.49–3.55	<0.001*	1.24	0.85–1.81	0.263	1.43	0.95–2.12	0.090

AOR: adjusted odds ratio; adjusted for all covariates: sex, age group, current education level, monthly income, GPA, past 30-day alcohol use, past 30-day cannabis use.

a Hosmer and Lemeshow test, step 1, χ^2^=11.642, df =8, p=0.168.

b Hosmer and Lemeshow test, step 1, χ^2^=9.838, df =8, p=0.277.

* p<0.05.

### Multivariable analyses

Six factors were identified as being associated with a higher likelihood of experiencing elevated levels of depression. These factors included being female (AOR=0.55; 95% CI: 0.45–0.67), attending a vocational school (AOR=0.59; 95% CI: 0.47–0.74), attending university (AOR=0.71; 95% CI: 0.56–0.90), drinking alcohol in the past 30 days (AOR=0.82; 95% CI: 0.68–0.99), using e-cigarettes in the past 30 days (AOR=0.81; 95% CI: 1.30–2.51), and using both e-cigarettes and cigarettes in the past 30 days (AOR=2.30; 95% CI: 1.49–3.55). Fewer factors were associated with increased odds of experiencing heightened levels of anxiety. These included being female (AOR=1.46; 95% CI: 1.23–1.73), attending vocational school (AOR=0.63; 95% CI: 0.52–0.76), smoking traditional cigarettes in the past 30 days (AOR=0.73; 95% CI: 0.55–0.96), and using e-cigarettes in the past 30 days (AOR=1.70; 95% CI: 1.24–2.32). Additional details are provided in Table 2.

## DISCUSSION

This study examined the correlation between current vaping and smoking behaviors and the presence of symptoms related to depression and anxiety among individuals aged 15–24 years in Thailand, a country where e-cigarettes are illegal. In the adjusted models, it was observed that those who were current e-cigarette users and those who used both e-cigarettes and traditional cigarettes were more likely to experience increased depression and anxiety symptoms. Interestingly, current smokers were found not to be associated with depression and have a lower likelihood of experiencing anxiety. The findings related to e-cigarette use align with those from high-income countries where regulations on e-cigarette products are less stringent^18^.

The most probable explanations for these observed connections may be attributed to the biological impact of nicotine on psychological processes. Nicotine is recognized for its capacity to influence mood and potentially affect neurotransmitter systems, thereby potentially triggering the release of chemicals like dopamine and norepinephrine. These biochemical actions can result in heightened alertness and temporary sensations of relaxation^9^. Conversely, as the effects of nicotine diminish, some individuals may encounter increased feelings of anxiety and depression, potentially contributing to a cycle of nicotine dependence^9^. However, it is essential to note that the precise mechanisms that underlie any potential association between e-cigarette use and depressive disorders remain not fully comprehended.

Moreover, research that concentrates on youth and adolescents has suggested that the use of e-cigarettes may be linked to an elevated likelihood of experiencing mental health issues within this demographic^19^. Young individuals might be more vulnerable to the impacts of nicotine, and exposure to nicotine during adolescence could potentially disrupt the development of their brains, rendering them more susceptible to the adverse consequences of nicotine on mood and anxiety^20^. Evidence also indicates that a significant proportion of young e-cigarette users are not well-informed about whether their e-liquid contains nicotine or have limited knowledge regarding the substance’s health effects^21^. Remarkably, consistent with global data indicating that one-third of adolescents aged 10–19 years are at risk of developing clinical depression, exceeding reported estimates for individuals aged 18–25 years^22^, this current research identified that being in a younger age group was associated with a higher likelihood of experiencing depression and anxiety. Accordingly, implementing restrictions on early-age engagement with nicotine-containing products has the potential to reduce the likelihood of such developments.

In addition to biological factors like younger age^23^, young individuals may also encounter added stressors associated with peer influence^23^, and social and academic functions^24^. These factors, for instance, could play a role in the development of depression. Understanding and addressing these intricate dynamics is essential in supporting the well-being of individuals in this population.

Another plausible interpretation of this connection involves a reverse or bidirectional causation^25^. In other words, it could be that individuals with pre-existing depression may be more inclined to resort to e-cigarettes as a means of coping or self-medicating, as opposed to e-cigarette use directly causing depression. This perspective aligns with the body of evidence indicating that stress relief is among the most frequently cited reasons for using e-cigarettes^26^. Notably, anxiety is not prominently identified as a motivator for the initiation of e-cigarette use. Furthermore, a review suggests an inconsistent association between anxiety and e-cigarette use^27^.

Dual behavior also appears to be significant, as this study demonstrates a connection between dual use and depression in the adjusted models. Individuals who concurrently use both e-cigarettes and traditional cigarettes may face an increased likelihood of experiencing mental health challenges as they are more likely to be exposed to toxicants produced by both products^28^. Nevertheless, the long-term consequences of dual-use on psychological distress remain a topic that lacks comprehensive understanding. Further investigation is warranted to ascertain whether the concurrent use of these products gives rise to distinct or intensified effects on mental health.

While established evidence supports the link between smoking and depression and anxiety among young people^29^, this study presents contrasting findings. As mentioned earlier, nicotine found in e-cigarettes might have a role in mental health concerns. It is important to note that e-cigarettes differ from traditional cigarettes in terms of their nicotine delivery mechanisms and compositions. Some e-cigarettes may deliver nicotine more effectively or in higher concentrations compared to their traditional counterparts^30^, which can potentially affect their impact on mental health. The chemical composition of e-liquids and the additives used in e-cigarettes also varies, potentially leading to distinct effects^31^.

Furthermore, other contributors may affect this link. In the present study, it is plausible that there is an overrepresentation of young adults, particularly undergraduates or college students, who have been smokers for an extended duration. This could explain the negative correlation between smoking cigarettes and anxiety in this study, as smokers may hold the belief that smoking alleviates stress, mainly because abstaining from smoking leads to stress, and cigarette use relieves withdrawal symptoms in smokers^32^. However, it is worth noting that quitting or reducing smoking has been associated with decreased levels of depression, stress, and anxiety while also contributing to improved mood and overall quality of life when compared to persistent smoking^33^. Advocating smoking cessation via anti-smoking campaigns may improve public education on smoking risks, alter attitudes and beliefs, boost intentions to quit, encourage cessation, and reduce smoking rates.

Societal and regulatory factors are likely to be significant contributors to the variations in how smoking and anxiety are perceived. In Thailand, the sale of traditional cigarettes is restricted to individuals aged ≥20 years, while e-cigarette products are entirely prohibited. Since e-cigarette products are illegal to purchase and use, this may result in hidden purchases and usage, potentially causing the users additional stress. On the other hand, smoking might be more socially tolerated and less associated with social stigma. In contrast to countries where e-cigarettes are available for purchase, such as the United States, where e-cigarettes have gained social acceptance^34^, these societal differences could influence how participants express their experiences related to anxiety.

Nonetheless, this explanation may be subject to debate, suggesting that a significant number of young individuals tend to view e-cigarettes as more appealing or less harmful than traditional cigarettes. This was observed in both the countries where they were legal^35^ or prohibited^36^. This underscores the complex and multifaceted nature of how perceptions regarding these products are shaped, which necessitates further exploration and analysis.

### Strengths and limitations

This study has several strengths. Firstly, it features a sizable and diverse sample of participants aged 15–24 years from Northern Thailand, selected through a rigorous stratified sampling method, enhancing the findings’ applicability to the broader population. Additionally, the study relies on standardized tools like PHQ-A and GAD-7 to gauge depression and anxiety symptoms, ensuring data consistency and comparability. Moreover, it achieved an impressive 97.8% response rate, signifying robust participant engagement and efficient data collection. Nonetheless, there are limitations to consider. First, the study’s cross-sectional design hinders establishing causal relationships between vaping/smoking and depression/anxiety. Secondly, reliance solely on self-reported questionnaires may underestimate the true prevalence of vaping/smoking and mental health issues. Incorporating objective measures of nicotine dependence and clinical diagnoses of depression and anxiety in future research would enhance the validity and accuracy of findings. Thirdly, the frequency of substance use in the last 30 days was not identified, which could introduce bias when seeking associations.

Furthermore, despite efforts to control for potential confounding variables, it is possible that some relevant factors were omitted or inadequately measured. Residual confounding could thus persist, potentially impacting the observed associations. Moreover, while our study aimed to control for potential confounding factors, including gender, we acknowledge that the disproportionate representation of females in our sample may still introduce some degree of bias. Lastly, the study’s conduct in a context where e-cigarettes are banned, may limit its generalizability to countries with different regulatory frameworks and vaping prevalence rates.

## CONCLUSIONS

This research offers valuable insights into the association between the use of e-cigarettes, the concurrent use of both e-cigarettes and traditional smoking, and the presence of depression among young individuals in Thailand. A comprehensive investigation is needed to determine causal relationships between these co-occurrences in countries where e-cigarettes are prohibited. To address these dual challenges effectively, a comprehensive approach should be adopted. It is vital to enforce existing tobacco control policies to mitigate the impact of traditional tobacco products and e-cigarettes on young people. Moreover, implementing a comprehensive program involving behavioral, communication, and educational interventions is of utmost importance. To prevent or reduce the development of clinical psychiatric distress, there is a need for effective interventions that encompass prevention, screening, support, and treatment.

## Data Availability

The data supporting this research are available from the authors on reasonable request.

## References

[1] World Health Organization. WHO global report on trends in prevalence of tobacco use 2000–2025. 2nd ed. World Health Organization; 2018. Accessed April 5, 2024. https://iris.who.int/handle/10665/272694

[2] Tehrani H, Rajabi A, Ghelichi-Ghojogh M, Nejatian M, Jafari A. The prevalence of electronic cigarettes vaping globally: a systematic review and meta-analysis. Arch Public Health. 2022;80(1):240. doi:10.1186/s13690-022-00998-w 36415010 PMC9682677

[3] Arrazola RA, Ahluwalia IB, Pun E, Garcia de Quevedo I, Babb S, Armour BS. Current Tobacco Smoking and Desire to Quit Smoking Among Students Aged 13-15 Years - Global Youth Tobacco Survey, 61 Countries, 2012-2015. MMWR Morb Mortal Wkly Rep. 2017;66(20):533-537. doi:10.15585/mmwr.mm6620a3 28542119 PMC5657874

[4] Kim J, Lee S, Chun J. An international systematic review of prevalence, risk, and protective factors associated with young people’s e-cigarette use. Int J Environ Res Public Health. 2022;19(18):11570. doi:10.3390/ijerph191811570 36141845 PMC9517489

[5] Wang G, Liu W, Song W. Toxicity assessment of electronic cigarettes. Inhal Toxicol. 2019;31(7):259-273. doi:10.1080/08958378.2019.1671558 31556766

[6] Hua M, Talbot P. Potential health effects of electronic cigarettes: a systematic review of case reports. Prev Med Rep. 2016;4:169-178. doi:10.1016/j.pmedr.2016.06.002 27413679 PMC4929082

[7] Javed S, Usmani S, Sarfraz Z, et al. A scoping review of vaping, e-cigarettes and mental health impact: depression and suicidality. J Community Hosp Intern Med Perspect. 2022;12(3):33-39. doi:10.55729/2000-9666.1053 35711397 PMC9195082

[8] Becker TD, Arnold MK, Ro V, Martin L, Rice TR. Systematic review of electronic cigarette use (vaping) and mental health comorbidity among adolescents and young adults. Nicotine Tob Res. 2021;23(3):415-425. doi:10.1093/ntr/ntaa171 32905589

[9] Rosenthal DG, Weitzman M, Benowitz NL. Nicotine addiction: mechanisms and consequences. International Journal of Mental Health. 2011;40(1):22-38. doi:10.2753/IMH0020-7411400102

[10] Sreeramareddy CT, Acharya K, Manoharan A. Electronic cigarettes use and ‘dual use’ among the youth in 75 countries: estimates from Global Youth Tobacco Surveys (2014-2019). Sci Rep. 2022;12(1):20967. doi:10.1038/s41598-022-25594-4 36470977 PMC9722706

[11] Phetphum C, Prajongjeep A, Thawatchaijareonying K, et al. Personal and perceptual factors associated with the use of electronic cigarettes among university students in northern Thailand. Tob Induc Dis. 2021;19(April):31. doi:10.18332/tid/133640 33897315 PMC8059432

[12] Cho MS. Factors associated with cigarette, e-cigarette, and dual use among South Korean adolescents. Healthcare (Basel). 2021;9(10):1252. doi:10.3390/healthcare9101252 34682932 PMC8535660

[13] Bauld L, MacKintosh AM, Eastwood B, et al. Young people’s use of e-cigarettes across the United Kingdom: findings from five surveys 2015-2017. Int J Environ Res Public Health. 2017;14(9):973. doi:10.3390/ijerph14090973 28850065 PMC5615510

[14] Choychod S, Hale III WW, Sarayuthpitak J, Tangdhanakanond K. A cross-sectional study on the prevalence of Thai adolescent depression. Kasetsart Journal of Social Sciences. 2023;44(2):509–516. doi:10.34044/j.kjss.2023.44.2.21

[15] Panyawong W, Pavasuthipaisit C, Santitadakul R. Validation of the Thai version of the Patient Health Questionnaire for Adolescents (PHQ-A) in adolescent psychiatric patients: validation of the Thai version of the PHQ-A. International Journal of Child Development and Mental Health. 2020;8(1):30-40. Accessed April 5, 2024. https://he01.tci-thaijo.org/index.php/cdmh/article/view/222261

[16] Spitzer RL, Kroenke K, Williams JB, Löwe B. A brief measure for assessing generalized anxiety disorder: the GAD-7. Arch Intern Med. 2006;166(10):1092-1097. doi:10.1001/archinte.166.10.1092 16717171

[17] Manea L, Gilbody S, McMillan D. Optimal cut-off score for diagnosing depression with the Patient Health Questionnaire (PHQ-9): a meta-analysis. CMAJ. 2012;184(3):E191-E196. doi:10.1503/cmaj.110829 22184363 PMC3281183

[18] Livingston JA, Chen CH, Kwon M, Park E. Physical and mental health outcomes associated with adolescent E-cigarette use. J Pediatr Nurs. 2022;64:1-17. doi:10.1016/j.pedn.2022.01.006 35121206

[19] Becker TD, Arnold MK, Ro V, Martin L, Rice TR. Systematic review of electronic cigarette use (vaping) and mental health comorbidity among adolescents and young adults. Nicotine Tob Res. 2021;23(3):415-425. doi:10.1093/ntr/ntaa171 32905589

[20] Yuan M, Cross SJ, Loughlin SE, Leslie FM. Nicotine and the adolescent brain. J Physiol. 2015;593(16):3397-3412. doi:10.1113/JP270492 26018031 PMC4560573

[21] Thongsutt T, Yusote C, Jubprang S, et al. Factors associated with knowledge and attitude towards e-cigarettes among undergraduate students in Thailand: a cross-sectional study. Asian Pac J Cancer Prev. 2023;24(2):559-567. doi:10.31557/APJCP.2023.24.2.559 36853305 PMC10162620

[22] Shorey S, Ng ED, Wong CHJ. Global prevalence of depression and elevated depressive symptoms among adolescents: a systematic review and meta-analysis. Br J Clin Psychol. 2022;61(2):287-305. doi:10.1111/bjc.12333 34569066

[23] Cruz ELDD, Martins PDC, Diniz PRB. Factors related to the association of social anxiety disorder and alcohol use among adolescents: a systematic review. J Pediatr (Rio J). 2017;93(5):442-451. doi:10.1016/j.jped.2017.05.001 28579354

[24] de Lijster JM, Dieleman GC, Utens EMWJ, et al. Social and academic functioning in adolescents with anxiety disorders: a systematic review. J Affect Disord. 2018;230:108-117. doi:10.1016/j.jad.2018.01.008 29407534

[25] Lechner WV, Janssen T, Kahler CW, Audrain-McGovern J, Leventhal AM. Bi-directional associations of electronic and combustible cigarette use onset patterns with depressive symptoms in adolescents. Prev Med. 2017;96:73-78. doi:10.1016/j.ypmed.2016.12.034 28024859 PMC5510594

[26] Han G, Son H. A systematic review of socio-ecological factors influencing current e-cigarette use among adolescents and young adults. Addict Behav. 2022;135:107425. doi:10.1016/j.addbeh.2022.107425 35908319

[27] Garey L, Olofsson H, Garza T, Shepherd JM, Smit T, Zvolensky MJ. The role of anxiety in smoking onset, severity, and cessation-related outcomes: a review of recent literature. Curr Psychiatry Rep. 2020;22(8):38. doi:10.1007/s11920-020-01160-5 32506166

[28] Pisinger C, Rasmussen SKB. The health effects of real-world dual use of electronic and conventional cigarettes versus the health effects of exclusive smoking of conventional cigarettes: a systematic review. Int J Environ Res Public Health. 2022;19(20):13687. doi:10.3390/ijerph192013687 36294263 PMC9603628

[29] Bataineh BS, Wilkinson AV, Sumbe A, et al. The association between tobacco and cannabis use and the age of onset of depression and anxiety symptoms: among adolescents and young adults. Nicotine Tob Res. 2023;25(8):1455-1464. doi:10.1093/ntr/ntad058 37042355 PMC10347972

[30] Voos N, Goniewicz ML, Eissenberg T. What is the nicotine delivery profile of electronic cigarettes? Expert Opin Drug Deliv. 2019;16(11):1193-1203. doi:10.1080/17425247.2019.1665647 31495244 PMC6814574

[31] DeVito EE, Krishnan-Sarin S. E-cigarettes: Impact of e-liquid components and device characteristics on nicotine exposure. Curr Neuropharmacol. 2018;16(4):438-459. doi:10.2174/1570159X15666171016164430 29046158 PMC6018193

[32] Grunberg NE. A neurobiological basis for nicotine withdrawal. Proc Natl Acad Sci U S A. 2007;104(46):17901-17902. doi:10.1073/pnas.0708964104 17989218 PMC2084266

[33] Taylor G, McNeill A, Girling A, Farley A, Lindson-Hawley N, Aveyard P. Change in mental health after smoking cessation: systematic review and meta-analysis. BMJ. 2014;348:g1151. doi:10.1136/bmj.g1151 24524926 PMC3923980

[34] Berg CJ, Stratton E, Schauer GL, et al. Perceived harm, addictiveness, and social acceptability of tobacco products and marijuana among young adults: marijuana, hookah, and electronic cigarettes win. Subst Use Misuse. 2015;50(1):79-89. doi:10.3109/10826084.2014.958857 25268294 PMC4302728

[35] Sharma A, McCausland K, Jancey J. Adolescent’s health perceptions of e-cigarettes: a systematic review. Am J Prev Med. 2021;60(5):716-725. doi:10.1016/j.amepre.2020.12.013 33775514

[36] Lozano P, Arillo-Santillán E, Barrientos-Gutíerrez I, Reynales Shigematsu LM, Thrasher JF. E-cigarette social norms and risk perceptions among susceptible adolescents in a country that bans e-cigarettes. Health Educ Behav. 2019;46(2):275-285. doi:10.1177/1090198118818239 30606077 PMC6438717

